# Polymer photonic crystal membrane for thermo-regulating textile

**DOI:** 10.1038/s41598-020-66731-1

**Published:** 2020-06-17

**Authors:** Salim Assaf, Mohamed Boutghatin, Yan Pennec, Vincent Thomy, Alexander Korovin, Anthony Treizebre, Michèle Carette, Abdellatif Akjouj, Bahram Djafari-Rouhani.

**Affiliations:** Institute of Electronic, Microelectronic and Nanotechnology (IEMN), UMR 8520, University of Lille, Villeneuve d’Ascq, France

**Keywords:** Materials science, Materials for optics, Photonic crystals

## Abstract

We study numerically the absorption and scattering properties of a polymer photonic membrane to thermoregulate the human body microclimate which corresponds to the area between the skin and a textile. We first show that the structuration of the absorbing photonic membrane with air holes leads to a modulation of the optical spectrum in the Mid-Infrared range. Indeed, we show that the membrane is able to modulate the transmission amplitude by 28% in benefit or deficit of both the absorption and reflection. We then studied the thermal balance between the human body and the surrounding environment through the photonic membrane. We found that, compared to a regular membrane, the photonic crystal structure behaves as a heating component that offers the possibility to reduce the temperature of the room up to +1 °C. The membrane is flexible, low cost, 3D-printable, free of metallic particles, and can easily be added to usual textiles.

## Introduction

For the past ten years, photonic nanostructures have represented a paradigm for the control of thermal radiations, offering a panel of exciting properties for energy applications^1,2^. Because of their abilities to control and manage electromagnetic waves at the Mid-Infrared (Mid-IR) wavelength scale, photonic nanostructures demonstrate their ability to manage thermal radiations properties in a way drastically different from conventional thermal emitters. Indeed, photonic crystal performances authorize to overtake the constraints of usual thermal emitters^3^ toward coherence, narrowband emission, polarization, directionality… The modification of Planck blackbody radiation by photonic band gap materials has been discussed, based on either simple photonic multilayered structures^4^ or more complex 1D^5,6^, 2D^7,8^ and 3D^9^ ones, thus making the demonstration of the enhancement, suppression and selectivity of thermal emission based on the photonic crystals properties. The fundamental advances in controlling thermal radiation led to different applications in the energy domain, as thermophotovoltaic devices that convert sunlight into thermal emission using hot absorber-emitter^10^. Although, the concept of daytime radiative cooling has been introduced with the objective to cool passively terrestrial structures by the use of a broad band photonic mirror for solar light, then emitting strongly in the Mid-IR within the atmospheric transparency window^11,12^.

More recently, another field of application has appeared in the thermal radiation control, with the introduction of photonic nanostructures in textiles for personal thermoregulation. The main motivation comes from the reduction of the building energy consumption of Heating, Ventilation and Air-Conditioning (HVAC) systems by locally providing heating or cooling in the human body close environment. To this end, personal thermoregulation photonic textiles have been proposed with the objective to obtain specific spectral properties. For personal heating textiles^13,14^, integration of metallic nanowires or particles embedded in polyethylene or cotton matrices has been proposed. The heating production is then generated from the human body radiation supplementary reflection that can even being increased by adding Joule heating to complement the passive insulation. For cooling purpose, recent papers have proposed different fibers^15–18^ or membranes^19–21^ fabrics to increase IR transparency. In the fiber configuration, Tong *et al*.^15^ proposed an infrared-transparent-visible-opaque-fabric (ITVOF) made of synthetic polymer fibers with intrinsically low IR absorbance and structured fibers to maximize IR transparency and visible opaqueness. For membranes fabrics, Hsu *et al*.^19^ develop experimentally a textile that promotes effective radiative cooling composed of nanoporous polyethylene (PE), transparent to Mid-IR human body radiations. The dual functionalities (cooling and heating) within a same textile has also been proposed in two different ways. First, Hsu *et al*.^22^ investigated a textile for human body radiation using a passive bilayer thermal emitter embedded inside an IR-transparent nano-PE that can perform both radiative heating and cooling using the same non-symmetric piece of textile. Second, reversible humidity sensitive clothing for personal thermoregulation was also proposed using shape memory polymer^23^. This smart textile has been designed to reversibly adapt the thermal insulation functionality, thus permitting the air flow and reducing the humidity level and the apparent temperature. Recently, Zhang *et al*.^24^ have proposed a dynamic control the Mid-IR in reaction of the relative humidity of the underlaying skin by coating triacetate-cellulose bimorph fibers with a thin layer of carbon nanotubes.

The personal thermoregulation appears as an exciting scientific challenge to investigate, especially in the energy efficiency context. Many subjective parameters are attached to the thermoregulation of the human body linked to the apparent temperature and personal resentment^25^. Therefore, a tremendous effort is still necessary to develop smart wearable thermoregulating textiles to meet user demand for better thermal comfort. Until now, the previous papers have reported on transparent polymers (as PE) for cooling or on the introduction of metallic particles for heating effects. Complex geometry or properties for the dual heating and cooling purposes has been also proposed. In the present paper, we aim to propose a simple micro-structured photonic membrane for the heating functionality. Our purpose is to take advantage of the modulation of the electromagnetic waves at the Mid-Infrared (Mid-IR) to increase the human body temperature. This is done through the structuration of the membrane, as compared to the regular structure. To reach this objective, we propose to take benefit of the absorption properties of the polymer together with the scattering properties of the photonic membrane to thermoregulate the temperature of the membrane. We chose the benzocyclobutene (BCB) for the demonstration but the results can be easily extended to every polymer containing such an absorption in the Mid-IR range.

In the first section of the paper we investigate the optical properties of the polymer membrane, considering the effect of its structuration on the reflection/transmission/absorption coefficients in the Mid-IR range, and analyze the origin of the specific features that occur. In the second part, we define and analyze the thermal balance between the human body and the indoor environment through the photonic membrane, considering the radiation, convection and conduction mechanisms.

## Optical properties of the BCB polymer membrane

### Model and method

**Figure 1:**
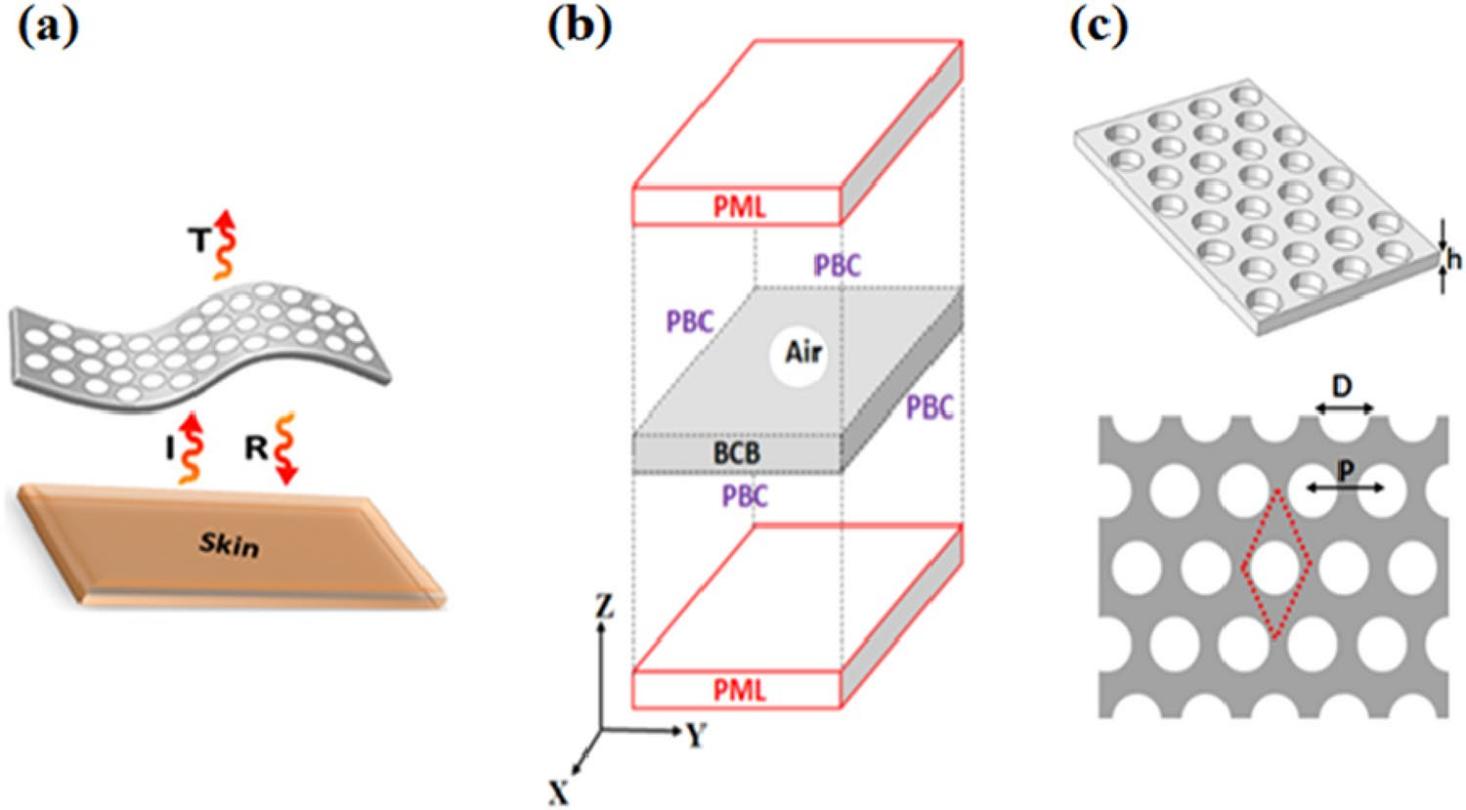
(**a**) Schematic representation of the polymer membrane textile under human body radiation from the skin where (I), (R) and (T) represent respectively the incident, reflection and transmission coefficients of the electromagnetic waves. (**b**) Elementary unit cell used for the FE calculations with perfect matching layers (PML) and periodic boundary conditions (PBC). (**c**) 3D and in-plane view of the polymer membrane of thickness *h*, drilled with a triangular array of air holes with period *P* and diameter *D*.

The investigation of the properties of a photonic membrane in the Mid-IR has been done numerically with the help of the Finite Element (FE) method. As seen schematically Fig. 1(a), we consider an incident electromagnetic waves (I) radiating in air from the human body skin and normally interacting with the photonic polymer membrane. All calculations have been done considering the polarization (E_y_, H_x_) of the incident light. Nevertheless, due to the honeycomb structuration of the photonic crystal array, the results can be extended to the other polarization. We record the transmission (T) and reflection (R) coefficients and then deduce the absorption coefficient following the equation A = 1 − R − T. The source and the detector are placed in air, respectively before and after the membrane. The structure of the elementary 3D unit cell used for the FE calculation is shown in Fig. 1(b). The incident source is a plane wave generated in the air medium which propagates along the z-direction, perpendicularly to the periodic structure. Along the x and y direction, periodic boundary conditions (PBC) are applied on each side of the unit cell to build the periodic structure. As the structure is supposed infinite along the z direction, perfect matching layers (PMLs) are applied at the finite boundaries of the box, along z, in order to avoid any reflections of outgoing waves. All calculations have been performed on a periodic triangular array of holes in the membrane. The geometrical parameters involved in the study are the lattice parameter *P*, the hole’s diameter *D*, and the thickness of the membrane *h* (Fig. 1(c)).

**Figure 2:**
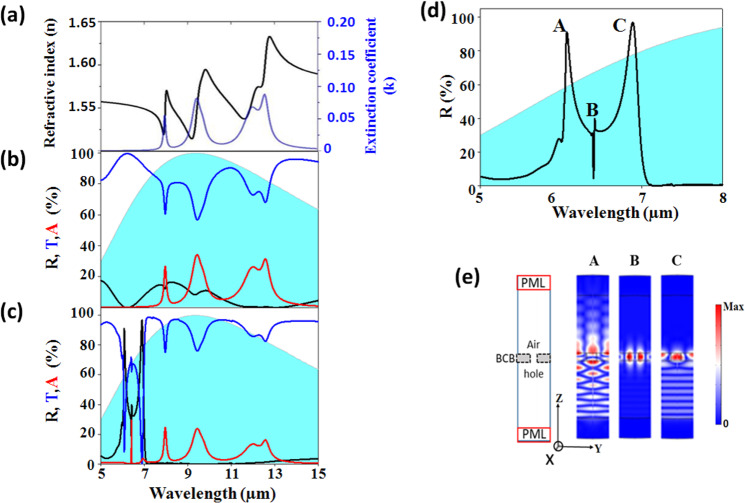
(**a**) Representation of the refractive index components (real *(n)* in black and imaginary *(k)* in blue) in the Mid-IR range [5-15] µm. (**b, c**) Reflection (black), transmission (blue) and absorption (red) spectra for the (b) non-structured and (c) structured BCB membrane with the geometrical parameters: *P* = 7.0 µm, *D* = 5.5 µm, *h* = 4.0 µm. (**d**) Zoom of the reflection curve in the wavelength range [5, 8] µm. The blue hatched area represents the black body emissivity of the human body, calculated from the Planck law at 34 °C. (**e**) *(Left)* Schematic representation of the unit cell in the *(y, z)* plane. The plane wave is launched from the bottom of the unit cell. *(Right)* Snapshots of the modulus of the electric field E, at the wavelength *λ*_A_ = 6.08 µm (A), *λ*_B_ = 6.41 µm (B) and *λ*_C_ = 6.88 µm (C).

We aim to develop a photonic crystal membrane that can modulate the Mid-IR radiations of the human body radiations. To reach the objective, we propose the study of a Benzocyclobutene (BCB) membrane, currently used in microelectronic manufacturing processes. As seen Fig. 2(a), the optical characterization of the BCB membrane, shows a variation of the real *(n)* and imaginary *(k)* part of the refractive index in the Mid-IR. Because of its non-polar chemical structure, its average refractive index of 1.57 makes it a good candidate to ensure a sufficient refractive-index contrast with air together with an absorption in the frequency range of interest (see Supp. Information 1 for more details).

### Spectral analysis of the photonic membrane in the Mid-IR

We first present Fig. 2(b), as a reference, the reflection, transmission and absorption coefficient spectra of a non-structured BCB membrane, having a thickness of *h* = 4.0 µm. At wavelengths close to 8.0 µm, 9.5 µm and 12.0 µm, several dips appear in the transmission spectrum (blue line), corresponding to absorption peaks in the spectrum (red line). These peaks are directly linked to the extinction coefficient of the refractive index of the BCB (Fig. 2(a)) and are thus attributed to the absorption of the polymer. One can note that all peaks appear in the human body emittance, calculated from the Planck law at the skin temperature of the human body T_s_ = 34 °C, represented within the blue hatched area. A small reflection (black curve), less than 20%, is recorded with a maximum at 8.5 µm and a zero at 6.0 µm. This modulation corresponds to Fabry-Perot oscillations through the BCB membrane of finite thickness.

We now consider the structured membrane, following a triangular array of air holes, with the geometrical parameters *P* = 7.0 µm, *D* = 5.5 µm, and *h* = 4.0 µm. Figure 2(c) shows the corresponding calculation of the reflection (black), transmission (blue) and absorption (red) spectra. We first find the absorption peaks of the BCB polymer, close to 8.0 µm, 9.5 µm and 12.0 µm, described previously. Qualitatively, it can be seen that the average level of transmission increases to the detriment of the reflection and absorption. Then, one can see the occurrence of new features appearing at low wavelengths, between 6.0 µm and 7.5 μm. To get a higher wavelength resolution, the reflection spectrum is magnified in the Fig. 2(d). We can remark that the high reflection occurs at two main peaks, at 6.08 µm and 6.88 µm, and a small one at 6.41 µm in the middle. To understand their origin, we performed calculations of the modulus *E* of the electric field at the corresponding wavelengths (Fig. 2e). One can see that the input signal, launched from the bottom, does not transmit through the membrane. In each case, the origin comes from the excitation of a stationary mode which belongs to the BCB membrane and couple to the incident wave. The two high reflection peaks (A and C) correspond to modes spread over the BCB membrane and are attributed to guided modes in the plate: one is antisymmetric (*λ*_A_ = 6.08 µm) and the other is symmetric (*λ*_C_ = 6.88 µm) with respect to the mid-plane of the membrane. The small peak in the middle (B) corresponds to an antisymmetric mode strongly confined inside the air holes. One can notice that the interaction of this localized mode with the incident wave propagating in the continuum, gives rise to a peak of asymmetric shape in the reflection spectrum. Such wave interaction phenomenon, known as a Fano resonance, is widely reported in the literature^26,27^

### Tuning of the spectral coefficients in the Mid-IR

The objective is now to tune the spectral coefficients in the Mid-IR range. To reach this objective, we proceed to a systematic calculation of the R, T and A coefficients as a function of the geometrical parameters of the photonic membrane. We showed that the reflection, transmission and absorption curves are affected by the structuration of the membrane, more particularly in the lower part of the emissive spectrum. To see the impact of the geometric parameters on the emissivity of the human body, we repeat the same calculations when all the geometrical dimensions of the membrane are multiplied by a scaling factor *α*_*i*_, where *i* = 1 corresponds to the geometrical parameters chosen in the previous section as a reference. Indeed, such a homothetic variation of the geometrical parameters, following a scale law, induces a similar scaling in the wavelength of the radiation effect of the membrane, thus emerging inside the human body emissivity. The supplementary information 2 reports the set of parameters associated to the scaling factors *α*_*i*_.

**Figure 3:**
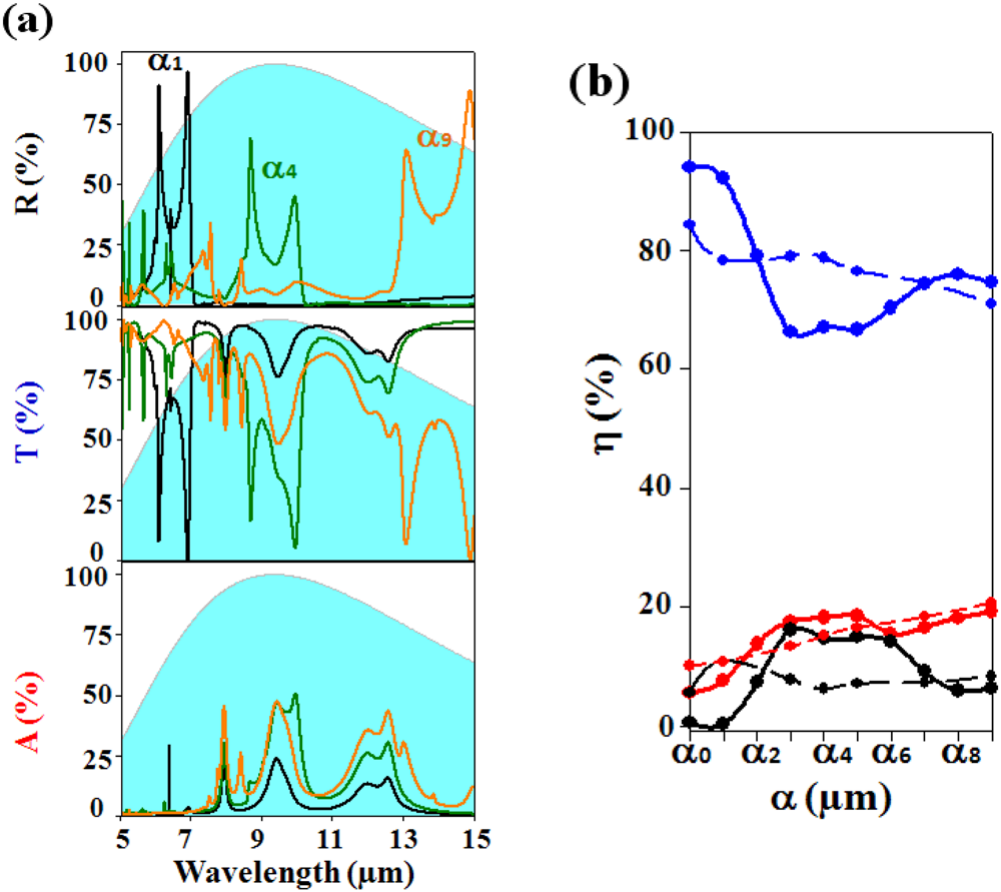
(**a**) Evolution of the reflection (R), transmission (T) and absorption (A) coefficients as a function of the wavelength in the Mid-IR for three photonic membranes, designed following the scaling factors *α*_1_, *α*_*4*_ and *α*_9_. (**b**) Evolution of the efficiency coefficient *η*, associated to the reflection (black), transmission (blue), absorption (red) responses of the non-structured (dashed lines) and structured (solid lines) BCB membrane.

The calculated reflection, transmission and absorption coefficients are shown in Fig. 3(a) for three scaling factors *α*_1_, *α*_4_ and *α*_9_. In the reflection spectrum, we found that, increasing the size of the geometrical parameters leads to the shift of the three reflective features A, B and C, towards higher wavelengths. In the transmission and absorption spectra, one can qualitatively follow the signature of the reflective modes, that fully cross the human body emissivity, and drastically affects both spectra.

To get a quantitative estimation of these behaviors, we defined an efficiency coefficient, *η*, corresponding to the integration of the R, T and A coefficients over a defined wavelength range corresponding to the human body emissivity. Such a coefficient is commonly used for the estimation of photovoltaic solar cells efficiency^28^. This coefficient is explained through the formula:

1 $$\eta =\frac{{\int }_{{{\boldsymbol{\lambda }}}_{{\boldsymbol{\min }}}}^{{{\boldsymbol{\lambda }}}_{{\boldsymbol{\max }}}}{{\boldsymbol{E}}}_{\lambda }.{{\boldsymbol{\chi }}}_{\lambda }.{\boldsymbol{d}}{\boldsymbol{\lambda }}}{{\int }_{{\lambda }_{{\boldsymbol{\min }}}}^{{\lambda }_{{\boldsymbol{\max }}}}{{\boldsymbol{E}}}_{\lambda }{\boldsymbol{d}}{\boldsymbol{\lambda }}}$$

where *E*_λ_ is the human body emissivity and χ_λ_ is one of the R, T or A coefficients at the wavelength *λ*. The integration will be done over the wavelength interval [*λ*_*min*_ = 7.5 µm – *λ*_*max*_ = 11.5 µm] that cover 70% of the human body emissivity with a maximum at *λ* = 9 µm.

Figure 3(b) reports the evolution of the efficiency coefficient *η* for the reflection (black solid line), transmission (blue solid line) and absorption (red solid line) as a function of the scaling factors *α*_*i*_. One can see that, increasing *α*_0_ to *α*_3_, the transmission drops by 28%, i.e. from 94% to 66% in benefit of the reflection and absorption which increase respectively by 16% (from 0.5% to 16%) and 12% (from 5.5% to 17.5%). The efficiency coefficient is then almost constant until the geometrical parameters reach *α*_6_ where the transmission increases again. As demonstrated previously, such tunability of the efficiency coefficients is directly related to the occurrence of the reflective peaks A, B and C over the wavelength interval. The tunability has been compared with the one of a non-structured membrane of same thickness (dashed lines). We clearly see that the structuration of the membrane made possible the tunability of the transmission coefficient, from transparent to opaque in the Mid-IR, by increasing the geometrical factor *α*_*i*_. We then obtained a photonic membrane with both a transparency (*α*_*i*_ ≤ *α*_1_) and an opacity (*α*_*i*_ > *α*_3_), higher by respectively ≈14% and ≈13% compared to an unstructured membrane.

**Figure 4:**
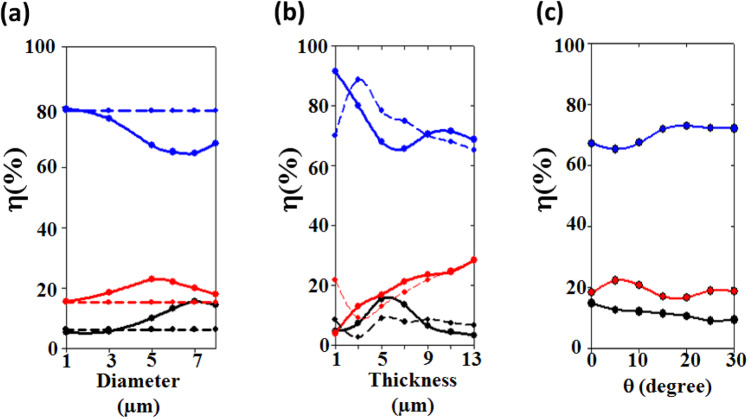
Evolution of the reflection (black), transmission (blue), and absorption (red) efficiency coefficients *η* in the range [7.5–11.5] µm, as a function of: (**a**) the diameter of air holes, keeping constant the period *P* = 10 µm, and the thickness *h* = 5.7 µm of the BCB membrane, (**b**) the thickness of the membrane for *P* = 10 µm, *D* = 7.9 µm, and (**c**) the incident angle *θ* of the incoming human body radiation for the scaling factor *α*_4_. The solid (resp. dashed) lines correspond to the structured (resp. non structured) responses.

Moreover, these tunabilities are supported by a complementary calculation where we change one geometrical parameter at a time while keeping the two others constant. In Fig. 4(a), the period *P* = 10 µm and thickness *h* = 5.7 µm are kept constant while we investigate the effect of the variation in the diameter D. When the diameter of the holes is small we reproduce the behavior of a non-structured membrane which is 80% transparent to the human body radiation. For a diameter *D* = 7.9 μm, corresponding to the scaling factor *α*_4_, we recover a decrease of the transmission down to 66%. In between, the spectral coefficients can still be tuned, for instance we obtain an increased opacity of 15% for *D* = 7 μm. We have also investigated the effect of the thickness *h* of the membrane, keeping constant *P* = 10.0 μm and *D* = 7.9 μm (Fig. 4(b)). One can see that the minimum of transmission is obtained when the reflection is maximum, i.e. for *h* = 5.7 μm. Finally, the low transmission has been investigated as a function of the angle of incidence of the human body radiations for the scaling factor *α*_4_. The numerical results (Fig. 4(c)) reveals a robustness of the transmission opacity with respect to the incidence angle at least until 10 degrees which is acceptable with respect to the hypothesis of an incident plane wave coming from the human body radiation.

## Thermal investigation

The objective of this section is to examine the effect of the optical properties found in section 1 on the thermal flows between the human body and the surrounding environment, through the membrane, and to demonstrate the heating power of the structured membrane. The comparison is done with a non-structured equivalent membrane.

### Thermal balance analysis

**Figure 5:**
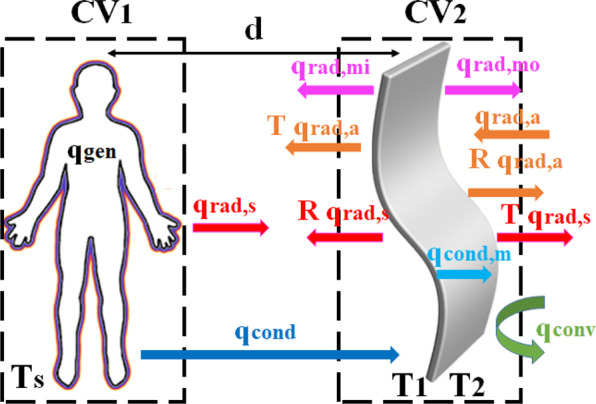
Schematic representation of the heat transfer model of the clothed human body to the surrounding environment, considering all radiative (rad.), conductive (cond.) and convective (conv.) mechanisms. The indices *s*, *a*, *mo* and *mi* respectively mean skin, ambient air, membrane inner and membrane outer surfaces. *d* is the distance between the human body and the membrane.

To quantify the impact and benefit of the photonic BCB membrane on personal thermoregulation, we rely on a one-dimensional steady-state heat transfer model analysis including radiative, conductive, and convective heat transfer mechanisms as shown in Fig. 5.

In the following, we provide a summary of the assumptions, input parameters, and analytical formula useful for the thermal calculation. Throughout this work we limit ourselves to a functionality of the membrane for everyday use, assuming that the human body is in a sedentary state, inside a room. As a consequence, uniform skin temperature and heat generation from the human body are considered. The air between the skin and the polymer membrane is assumed to be stationary, meaning that convective heat transfer will be neglected in this region. In the model, both skin and environment are assumed to be respectively an ideal blackbody emitter and absorber. The membrane will then partially reflect, transmit and absorb the incoming optical radiation, in support of the optical results obtained in section 1. We used Kirchhoff’s law and define the emissivity ε_m_(λ) of the membrane throughout its absorption coefficient, as:

2 $${\varepsilon }_{m}(\lambda )=A=1-R-T$$

where A, R and T are respectively the absorbance, reflectance and transmittance of the polymer membrane calculated in section 1.

The equation of the thermal balance can be defined considering two volumes of control, respectively embedding the human body (CV1) and the BCB membrane (CV2) (Fig. 5). By calculating the incoming and outgoing flows passing through these volumes, the energy balance at the CV1 and CV2 can be expressed as:

3 $${\text{q}}_{\text{gen}}-{\text{q}}_{\text{rad},\text{s}}(1-R)-{\text{q}}_{\text{cond}}+T.{\text{q}}_{\text{rad},\text{a}}+{\text{q}}_{\text{rad},\text{mi}}=0$$

4 $${\text{q}}_{\text{cond}}+A.{\text{q}}_{\text{rad},\text{s}}+A.{\text{q}}_{\text{rad},\text{a}}-[{\text{q}}_{\text{rad},\text{mi}}+{\text{q}}_{\text{rad},\text{mo}}+{\text{q}}_{\text{conv}}]=0$$

where q_gen_=73W.*m*^−2^ is the metabolic body heat production rate per unit area in a middle-aged man standing and relaxed, q_rad,s_ is the radiative heat flux from the skin, q_rad,a_ is the radiative heat flux from the ambient air, q_rad,mi_ is the radiative heat flux from the polymer membrane inner surface, q_rad,mo_ is the radiative heat flux from the polymer membrane outer surface, q_cond_ is the conductive heat flux in the air gap between the skin and the textile, q_conv_ is the convective heat flux from the membrane to the ambient air.

The conductive, convective, and radiative heat flux terms follow respectively the Fourier, Newton, and Stefan‐Boltzmann laws and take the following expressions:

5 $${\text{q}}_{\text{rad},\text{s}}=\sigma \cdot {\text{T}}_{\text{S}}^{4}$$

6 $${\text{q}}_{\text{rad},\text{a}}=\sigma \cdot {\text{T}}_{\text{a}}^{4}$$

7 $${\text{q}}_{\text{rad},\text{mo}}={\varepsilon }_{m}(\lambda ).\sigma .{\text{T}}_{2}^{4}$$

8 $${\text{q}}_{\text{rad},\text{mi}}={\varepsilon }_{m}(\lambda ).\sigma .{\text{T}}_{1}^{4}$$

9 $${\text{q}}_{\text{cond}}={k}_{air}\frac{{\text{T}}_{\text{S}}-{\text{T}}_{1}}{d}$$

10 $${\text{q}}_{\text{conv}}={h}_{c}({\text{T}}_{2}-{\text{T}}_{\text{a}})$$

where T_S_ is the temperature of the skin (T_S_ = 34 °C), T_a_ is the temperature of the ambient air, T_1_ and T_2_ are respectively the temperatures of the inner and outer surfaces of the membrane. *k*_*air*_ is the thermal conductivity of air (*k*_*air*_ = 0.027 W.m^−1^.K^−1^), *h*_*c*_ is the convective heat transfer coefficient (*h*_*c*_ = 3.0 W.m^−2^.K^−1^), and σ is the Stefan-Boltzmann constant (σ = 5.6710^−8^ W.m^−2^.K^−4^). Note that the convective heat transfer coefficient *h*_*c*_ can be adapted to simulate different environmental conditions as, for example, the air circulation within the room (not considered here). Additionally, the air gap thickness, *d*, has been fixed to *d* = 2 mm and can be adapted as well.

To these two Eqs. (3) and (4), we have to add a third equation describing the heat transfer (conduction) mechanism through the membrane, q_cond,m_. From the temperature profile within the textile demonstrated in ref. ^16^ (see SI, Eq. S22), we are able to define a relation between the temperatures T_1_ and T_2_ respectively at the inner and outer faces of the membrane as:

11 $${T}_{2}=\frac{h}{2{k}_{m}}\{{\varepsilon }_{m}\sigma {({T}_{1})}^{4}+{\varepsilon }_{m}\sigma {({T}_{2})}^{4}-{\varepsilon }_{m}\sigma {T}_{s}^{4}-{\varepsilon }_{m}\sigma {T}_{a}^{4}\}-\frac{{k}_{air}h}{{k}_{m}d}({T}_{s}-{T}_{1})+{T}_{1}$$

where ${k}_{m}=0.2W.{m}^{-1}.{K}^{-1}$ is the BCB’s thermal conductivity^29^, *h* is the thickness of the membrane, and *d* is the thickness of the microclimate. Actually, due to very small thickness of our membrane, it is expected that the temperatures T_1_ and T_2_ will be almost the same as we shall see numerically in the next section.

### Determination of the BCB and ambient temperatures

The efficiency of the membrane is demonstrated by fixing the temperature of the skin, typically around T_s_ = 34 °C and to determine the unknown temperatures T_1_, T_2_, and T_a_ from the resolution of Eqs. (3), (4) and (11).

**Figure 6:**
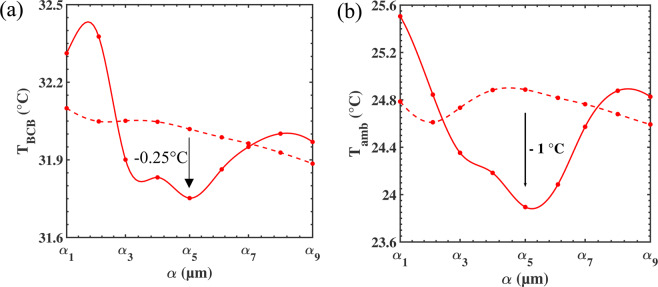
Evolution of (**a**) the temperature of the membrane T_BCB_ = T_1_ = T_2_ and (**b**) the ambient temperature as a function of the scaling factor α_i_ for a structured (solid lines) and non-structured (dashed lines) membrane.

In Fig. 6(a,b), we show the evolution of the temperatures T_1_, T_2_ and T_a_ as a function of the scaling factor α_i_. To estimate the effect of the structuration of the membrane, we compare the results (solid lines in Fig. 6) with those of the non-structured membrane of equivalent thickness (dashed lines). First, one can notice that the inner and outer temperatures of the membrane are exactly the same, reported as T_BCB_ in Fig. 6a. This is due to the extreme thinness of the membrane, typically smaller than 10 µm. Then, when the scaling factor increases, the temperature varies from 32.4 °C to 31.75 °C, reaching a minimum for α_i_ = α_5_. This modulation of temperature follows the variation in transmission (see Fig. 3b) realized in benefit of the reflection and absorption of the membrane. Finally, if we consider the geometrical parameters of the membrane associated to α_5_, one can see that the temperature is lower in the structured membrane as compared to the non-structured membrane of equivalent thickness by about 0.25 °C. This means that, to keep the skin at the comfort temperature of 34 °C, a lower temperature is needed in the structured membrane as compared to the unstructured one. Therefore, as we shall see from Fig. 6b, a lower ambient temperature will be sufficient to provide the same comfort.

Indeed, another way to demonstrate the efficiency of the structuration is to represent the ambient temperature calculated from Eqs. (3), (4) and (11) as a function of the scaling factor (Fig. 6b). This representation allows to compare the necessary temperature of the room to keep the thermal comfort of the human body. For the design α_5_, the calculated ambient temperature can be up to +1 °C lower for the structured membrane as compared to the non-structured one. This property offers the opportunity of maintaining the individual comfort while reducing the external energy supply of the room.

**Figure 7:**
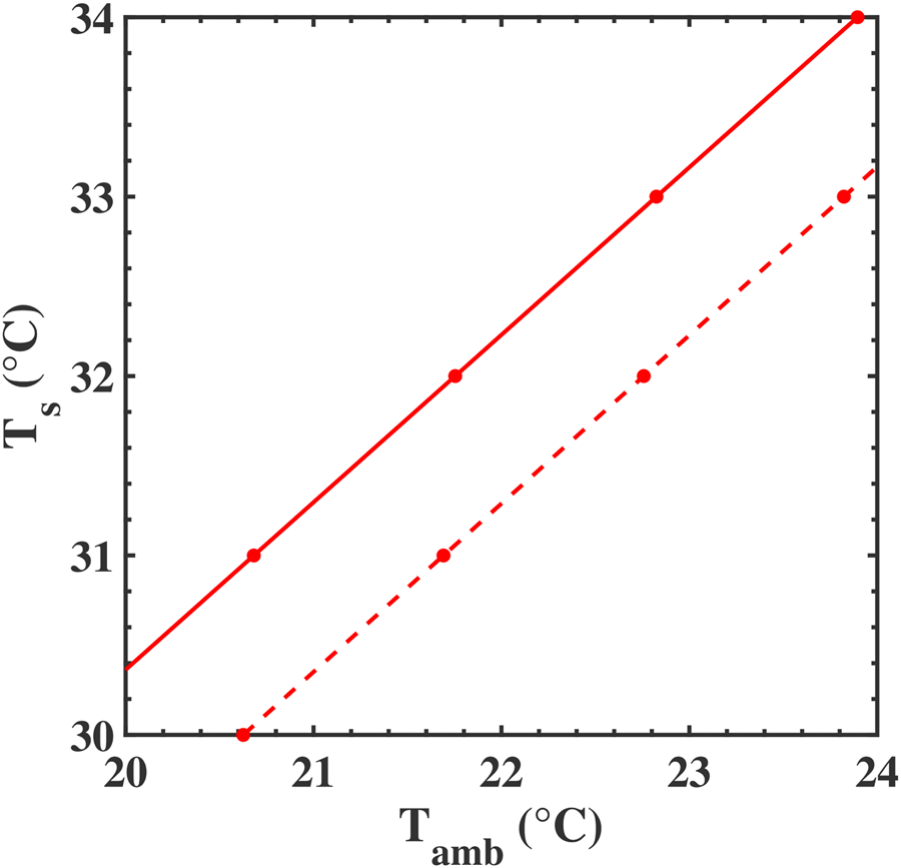
Evolution of the skin temperature as a function of the ambient temperature for the structured (solid line) and unstructured (dashed line) membrane (from SI, table 3).

We also calculated the set of temperatures (T_1_, T_2_, T_a_) for different skin temperatures slightly lower than 34 °C (see SI, table 3). Figure 7 shows the evolution of the skin temperature as a function of the ambient temperature for the structured and unstructured membrane. One can clearly see that the temperature of the skin is systematically 1 °C higher when the human body is clothed with the structured membrane. It demonstrates the heating capacity of the photonic membrane. A variation of −1 °C would represent a significant reduction in energy consumption, leading to economic and environmental benefits.

## Conclusion

We have demonstrated the thermoregulation properties of a photonic crystal membrane based on the modulation of the human body optical radiations in the Mid-IR range. We first study the transmission, reflection and absorption properties of the polymer membrane in the Mid-IR domain. The polymer under consideration is the Benzocyclobutene (BCB) which presents both an absorbance and sufficient refractive-index contrast with air to deal with the photonic crystal properties following a triangular array of air holes. We found that the structuration leads to new features in the spectral reflection, transmission and absorption spectra that have been identified as guided and localized modes of the photonic crystal membrane. We then introduced a quantitative coefficient to estimate the efficiency of the transmission of the human body emissivity through the photonic membrane. We found that, by scaling the geometrical parameters of the crystal, the transmission can be modulated up to 28% of the human body emissivity in the wavelength range [7.5, 11.5] µm.

We then coupled the optical properties with thermal balance applied between the human body and the surrounding environment, through the structured membrane. We found that for a set of geometrical parameters (*P* = 11 µm, *D* = 8.6 µm, *h* = 6.3 µm) the membrane behaves as a thermoregulator of the human body. We found that, compared to a non-structured regular membrane of equivalent thickness, the temperature of the room needs to be systematically 1 °C less when the human body is clothed with the structured membrane in order to get the same thermal comfort (T_s_ = 34 °C). We believe that such a simple photonic membrane offers a new opportunity for the thermal management of textile for everyday use, and targets one of the major domains in energy consumption. Additionally, the presence of pores drilling the membrane enables air permeability and can promote water-wicking.

## Supplementary information

Supplementary Information.
