# Supplementary material for: Polymer photonic crystal membrane for thermo-regulating textile
Source: Sci Rep. 2020 Jun 17;10:9855. doi: 10.1038/s41598-020-66731-1 (PMC7300027; doi:10.1038/s41598-020-66731-1)
Supplement: Supplementary file 1 — Supplementary Information. [file 41598_2020_66731_MOESM1_ESM.docx]

**Supplementary Information 1.**

In the calculations, the frequency-dependent complex permittivity of BCB, with real *(n)* and imaginary *(k)* components, obtained from ellipsometry measurements (data provided by the society Semilab), has been described by the Drude-Lorentz oscillator model with the help of the software PeakFit, version 4.1.2/2007 ([www.systat.com/products/PeakFit/](http://www.systat.com/products/PeakFit/)):

$\varepsilon_{DL}=\varepsilon_{\infty}+\sum_{i=1}^{5} \frac{n_{pi}^{2}}{n_{0i}^{2}-n^{2}+jn Г_{i}}$

Where $\varepsilon_{\infty}$ is the high frequency dielectric constant and$j=\sqrt{-1}$. $n_{pi}$, $n_{0i}$ and Г*_i_* are respectively, the plasma frequency of the oscillator, the resonant energy and the damping factor of the *i^th^* oscillator (see table 1).

Table 1. Physical parameters of the BCB photonic membrane

| $\varepsilon_{\infty}=2.3447$ | | | |
| --- | --- | --- | --- |
| *i^th^* oscillator | $n_{pi}$ *(cm^-1^)* | $n_{0i}$ *(cm^-1^)* | Г*_i_ (cm^-1^)* |
| 1 | 63.8886206 | 794.224391 | 25.6254127 |
| 2 | 101.666530 | 834.872232 | 65.9622864 |
| 3 | 46.0499229 | 1028.55930 | 37.4601652 |
| 4 | 120.316245 | 1060.78317 | 58.6347542 |
| 5 | 65.2296026 | 1256.51605 | 20.7377084 |

**Supplementary Information 2.**

Table 2. Set of parameters of the BCB photonic membrane following a scaling law indexed by *α_i_*.

| Scaling factor *α_i_* | Period *P* (µm) | Diameter *D* (µm) | Thickness *h* (µm) |
| --- | --- | --- | --- |
| * α−_1_* = 0.72 | 5.0 | 3.9 | 2.9 |
| *α__* = 0.86 | 6.0 | 4.7 | 3.4 |
| *α_1_* = 1.0 | 7.0 | 5.5 | 4.0 |
| *α_2_* = 1.14 | 8.0 | 6.3 | 4.6 |
| *α_3_* = 1.28 | 9.0 | 7.1 | 5.1 |
| *α_4_* = 1.42 | 10.0 | 7.9 | 5.7 |
| *α_5_* =1.56 | 11.0 | 8.6 | 6.3 |
| *α_6_* =1.7 | 12.0 | 9.4 | 6.9 |
| *α_7_* =1.84 | 13.0 | 10.2 | 7.4 |
| *α_8_* =1.98 | 14.0 | 11.0 | 8.0 |
| *α_9_* = 2.12 | 15.0 | 11.8 | 8.6 |

**Supplementary Information 3.**

The table 3 reports the temperatures (T_1_, T_2_, T_a_) calculated from eq. (2), (3) and (11) for different skin temperature lower than 34°C, for the unstructured *(unstruct.)* and structured *(struct.)* membranes.

| $T_{S}$ (°C) | 30 | | 32 | | 34 | |
| --- | --- | --- | --- | --- | --- | --- |
| *(Membrane)* | *(unstruct.)* | *(struct.)* | *(unstruct.)* | *(struct.)* | *(unstruct.)* | *(struct.)* |
| $T_{1}$ (°C) | 27.974 | 27.703 | 29.996 | 29.728 | 32.019 | 31.752 |
| $T_{2}$ (°C) | 27.973 | 27.702 | 29.996 | 29.727 | 32.018 | 31.751 |
| $T_{a}$ (°C) | 20.626 | 19.610 | 22.757 | 21.753 | 24.886 | 23.894 |
